# Multiexposure laser speckle contrast analysis system calibration limited by perfusion-dependent scattering on the skin

**DOI:** 10.1117/1.JBO.28.9.096006

**Published:** 2023-09-15

**Authors:** Tamás Smausz, Bence Kondász

**Affiliations:** University of Szeged, Department of Optics and Quantum Electronics, Szeged, Hungary

**Keywords:** skin perfusion, speckle imaging, laser speckle contrast analysis, light scattering

## Abstract

**Significance:**

Application of multiexposure speckle contrast imaging (MESI) methods for perfusion measurements can correct for the contribution of static scattering of the skin, at the expense of reduced temporal resolution as compared to classical single-exposure methods. Persistence of tissue scattering properties during the measurements could allow for an initial calibration and enhancement of the temporal resolution of the measurements.

**Aim:**

We aim to study the influence of the perfusion on the light scattering of the forearm skin and to use the obtained data for the enhancement of the temporal resolution.

**Approach:**

A wide range of skin perfusion states was induced while monitoring the changes in the dynamic range of the exposure-dependent contrast. Different measurement and evaluation methods were tested based on an initial MESI calibration followed by image recording with reduced number of exposure time values.

**Results:**

The changes in the skin perfusion can alter not only the contribution of the static scattering to the speckle images but also the short-exposure time contrast limit.

**Conclusions:**

The perfusion-dependent scattering of the skin can invalidate the precalibrations (e.g., β calibration) characterizing the combination of the given tissue and the measurement system.

## 1 Introduction

Since Fercher and Briers^1^ introduced laser speckle contrast analysis (LASCA), it has gained popularity as a low-cost method allowing real time, large area perfusion monitoring. The original theoretical formula,^1^ which gave a quantitative relationship between the contrast of the speckle images, the exposure time, and the correlation time, was further modified in order to increase the accuracy of the measurements.^2^^,^^3^ Considering that the contrast of the recorded speckle images for short-exposure times is smaller than 1, a correction factor β was introduced as follows:^3^

$$K{\left(T\right)}^{2}=\beta \frac{\tau^{2}}{2T^{2}}[\exp(\frac{-2T}{\tau })-1+\frac{2T}{\tau }],$$ (1)

where K(T) is the contrast of the image recorded at T exposure time and τ is the correlation time of the intensity fluctuation of the speckle images. For the asymptotic value corresponding to the infinitely short-exposure time $K{\left(0\right)}^{2}=\beta$, β is an overall characteristic of the optical imaging system, comprising the applied laser and the optical properties of the monitored tissue. The achievable contrast is influenced by the ratio between speckle size (depending on wavelength and optical settings) and pixel size, as well as by the depolarization of the light during scattering and polarization of light entering the camera.^4^ The contrast is also affected by the absorption of the sample^5^ and the degree of coherence of the scattered light. Since the coherence length of the laser source is finite, photons with different path lengths (depending on the optical properties of the tissue) will interfere on the sensor influencing the coherence and thus the contrast of the interference patterns.^4^^,^^6^ As a classical evaluation method, generally, β was obtained by measuring the contrast of the speckles recorded on a non-moving Teflon block, and then correlation times were calculated based on images recorded with a constant exposure time [single-exposure speckle imaging (SESI)]. Many applications of the LASCA involved measurements on skin in relation to diseases and burns.^7^^–^^10^ The application of polarizing optics can reduce the surface scattering^11^ and even make possible the imaging of skin microvascularization.^12^ However, in addition to the dynamic light scattering of the moving red blood cells (RBC), there is an intense volume scattering from the stationary components, which causes the asymptotic contrast value for long exposure time [K(∞)] to be much larger than zero. Since static scattering adversely affects the accuracy of data evaluation based on Eq. (1), several methods have been proposed to address this issue, which have significantly reduced the discrepancy between the results obtained on tissue phantoms consisting of microsphere emulsions covered with various scattering layers.^13^^–^^16^ In the method used by Zakharov et al.,^13^ after a β calibration, the static contribution was estimated by cross correlation of the consecutive speckle images. Recording of speckle images with a wide range of different exposure times [multiexposure speckle imaging (MESI)] allows the experimental measurement of the dependence of the contrast on the exposure time and the calculation of the correlation time using the K(T) equation, which takes into account the effects of β and the static scattering.^14^^,^^15^^,^^17^ In the MESI evaluation method proposed by Parthasarathy et al.,^14^ the correlation time and the dynamically scattered fraction of the light is obtained by fitting their K(T) curve to the contrast data; however, it also requires a precalibration of the system by determining the value of β. Smausz et al.^15^ proposed a simple semi-empirical formula to describe the relationship between the measured speckle contrast and the exposure time:

$$K(T{)}^{2}=P_{1}^{2}\frac{\tau^{2}}{2T^{2}}[\exp(\frac{-2T}{\tau })-1+\frac{2T}{\tau }]+P_{2}^{2},$$ (2)

where $P_{1}$ and $P_{2}$ are the weighting parameters. While $P_{1}^{2}$ scales the dynamic component of the $K{\left(T\right)}^{2}$ function, the $P_{2}^{2}$ static component comprises the time-independent spatial intensity variance caused by the static scattering layer and the noise of the experimental measurement. $P_{1}$ and $P_{2}$ [or the K(0) and K(∞)] together are the characteristics of the combination of the given optical system and the monitored tissue, where the asymptotic values of the contrast are $K(\infty )=P_{2}$ and $K(0)={\left(P_{1}^{2}+P_{2}^{2}\right)}^{1/2}$. This method does not require precalibration of the measurement system, as the $P_{1}$ and $P_{2}$ are obtained by continuous fitting of Eq. (2) to the contrast data. A disadvantage of the MESI method is that the reliable function fitting requires an exposure time range of about two orders of magnitudes, and the higher the number of different exposure time values is, the lower the temporal resolution of the perfusion data will be. We have to note that using high-speed (>1000  fps) cameras at the shortest exposure time and creating the longer exposure time images by adding up the required number of short-exposure images, the temporal resolution of the method can be significantly improved.^18^^,^^19^ However, the cost of such a system is much higher compared to a conventional MESI arrangement.

MESI contrast measurements on tissue phantoms showed that when the optical property was changed by covering purely dynamic microsphere emulsions with scattering layers, in addition to the increase of K(∞) (caused by the presence of static scattering), the value of K(0) also changed.^13^^–^^15^^,^^20^ If a significant change of K(0) occurs during the perfusion measurements, the initial β calibration of a measurement system may lose its validity. Our aim is to perform a systematic investigation on skin perfusion measurements focusing on the effect of different stimuli on the K(0) and K(∞) of the same area. Information on the magnitude of the changes within a series of measurements can help in optimizing the MESI measurements to enhance the temporal resolution or eventually allowing a (partial) calibration on the measured tissue. We applied stimuli often used in LASCA measurements, such as cooling of the skin,^21^^–^^24^ application of capsaicin,^17^^,^^21^^,^^25^^,^^26^ and occlusion.^27^^,^^28^

## 2 Materials and Methods

### 2.1 Experimental Setup

**Fig. 1:**
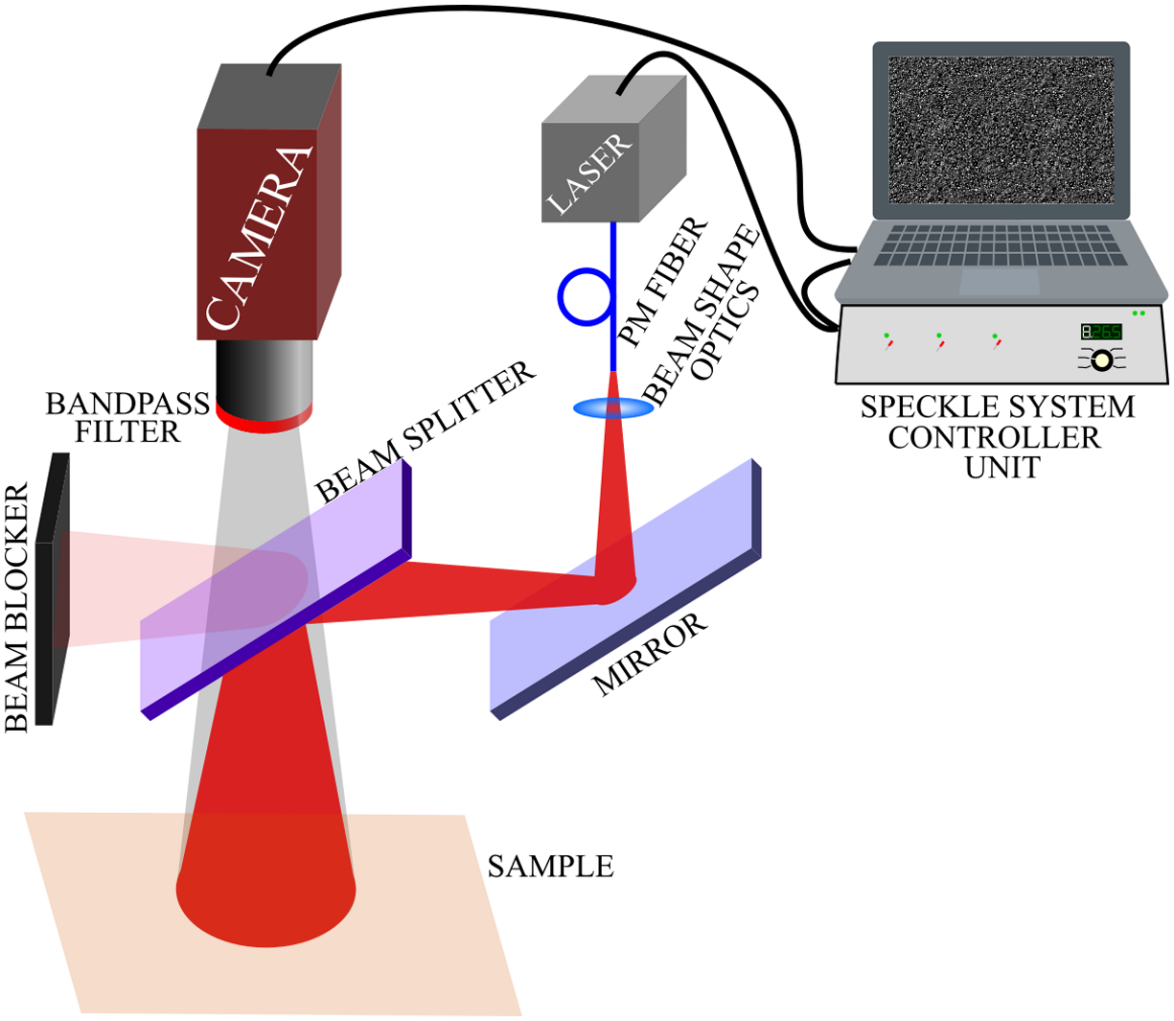
Experimental setup.

The experiments were performed using the setup shown in Fig. 1. The light source of the multiexposure LASCA system was volume holographic grating 785 nm wavelength-stabilized laser diode (Thorlabs LD785-SEV300) placed in a temperature-controlled mount. The current of the diode was controlled in switching mode, as presented in detail in Ref. 29. The laser light was guided by a polarization-maintaining single-mode optical fiber (Thorlabs PM780-HP) to the beam shaping optics. The speckle images were captured with a monochrome NIR enhanced CMOS camera (Allied Vision, Mako G-419B NIR, 5.5  μm×5.5  μm pixel size, 2048  px×2048  px, bit depth 12 bit, and global shutter mode) by 6 fps. The camera was equipped with a Computar M3514-MP (f=35  mm) objective with a spatial resolution of 6.5  μm/px and f/# was 16.

To avoid shadow effect, a coaxial system based on polarizing plate beam splitter (Thorlabs PBSW-780R) was used. The polarizing beam splitter minimizes the direct surface reflection and enhances the contribution of the depolarized volume scattering to the speckle image. To minimize ambient light, a bandpass filter (FB800-40) tuned to the wavelength of the laser light was used. The camera integration time was continuously 162 ms and the exposure time was set by the laser illumination as described in previous articles.^29^^,^^30^ Keeping the integration time at a constant value ensures that the sensor noise and its contribution to the speckle contrast remain constant. According to our measurements, the contribution of the noise to $K{\left(T\right)}^{2}$ is $∼2\times 10^{-3}$. In the experiment, exposure times of 1, 3, 9, 27, 81, and 162 ms were applied while background images were acquired periodically and subtracted from the speckle images to eliminate the contribution of ambient light. Image analysis, acquisition, and system control were performed by our software developed in the LabView (National Instrument) environment.

### 2.2 Determination of Fitting Parameters and Speckle Perfusion Data

The continuous MESI evaluation method was applied as follows. Images were recorded continuously by repeating the following exposure time sequence: background image, 1, 9, 3, 9, 27, 9, 81, 9, 162, and 9 ms. Image contrast was calculated on a 5  px×5  px sliding window. $P_{1}$ and $P_{2}$ fitting parameters [and thus the K(0) and K(∞)] were obtained by fitting the K(T) curve to the contrast values recorded at certain exposure times. For each exposure time, the contrast value was the running average of the last five corresponding images, $P_{1}$ and $P_{2}$ values were updated after the entire exposure time series was recorded. Based on the last known values of $P_{1}$ and $P_{2}$, the running average of 9 ms exposure time image (perfusion monitoring images) was used to calculate the correlation time. Since every second image was recorded with 9 ms exposure time, the perfusion data are updated five times more frequently than the $P_{1}$ and $P_{2}$ parameters. Tissue perfusion was characterized by the reciprocal value of the correlation time, which will be referred to as speckle contrast perfusion unit (SCPU) in this paper.

### 2.3 Methods

#### 2.3.1 Measurements on skin

The extreme values of the contrast curve and the variations of perfusion were investigated in human forearm skin. Measurements were made in an air-conditioned room after a 20 min acclimatization process. Different stimuli were applied to obtain a variety of perfusion magnitudes and optical properties of the tissue. These stimuli included: forearm arterial occlusion, local cooling, capsaicin treatment, and capsaicin treatment followed by occlusion, resulting in a range of flow rates and RBC concentrations. Arterial occlusion was applied on the upper arm by pumping the cuff of a blood pressure monitor to 200 mmHg. Cooling of the skin was achieved with a 25 mm diameter copper block. The block was cooled in icy water and then placed on the skin surface. It was removed after 5 min and blood flow measurements were then started. To achieve high perfusion rate, capsaicin cream was applied on the monitored skin surface. Measurements were performed 10 min after the application of the cream when the skin became red, and a nearly steady-state increased perfusion was obtained. Each stimulus was preceded by a reference (baseline) recording on the very same area against that the effects of the stimuli were compared. Data were obtained from measurement series on four persons, repeated three times on different days.

#### 2.3.2 Measurements on microsphere emulsion

Viscous microsphere emulsion with decreased Brownian motion and increased speckle correlation time (35 ms) was prepared in the following composition: 7  mg/ml of 3  μm polystyrene microspheres (monodisperse, microparticles GmbH) in water–glycerol (30 to 70 v/v%) mixture. Hydrostatic pressure driven flow of the emulsion through an opaque pipe (2.5 mm inner diameter and 4 mm outer diameter) with intense static scattering was monitored. To modify the scattering properties, one part of the pipe in the camera’s field of view was covered with a 50  μm thick polytetrafluoroethylene (PTFE) sheet.

## 3 Results and Discussions

**Fig. 2:**
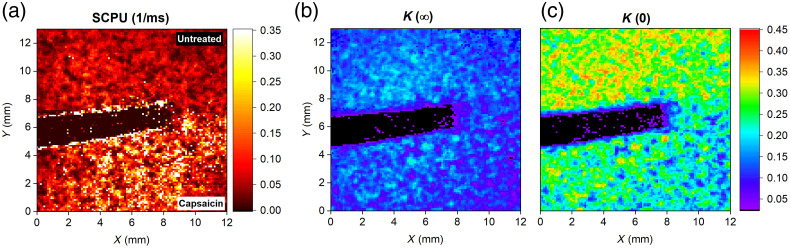
Map of (a) SCPU, (b) K(∞), and (c) K(0) values obtained over skin area partly treated with capsaicin. The black stripe corresponds to an adhesive tape marking the edge of the treated region.

For illustration, Fig. 2 shows the map of parameters (K(0), K(∞)) characterizing the dynamic range of K(T) curves and SCPU around the boundary of an area treated by capsaicin. The granular structure of the images can be attributed to skin inhomogeneity and uncertainties caused by frame-to-frame displacement of the monitored area. In order to avoid such evaluation errors, the forthcoming results were obtained using spatial averaging of the contrast values over an area of 10  mm×10  mm.

**Fig. 3:**
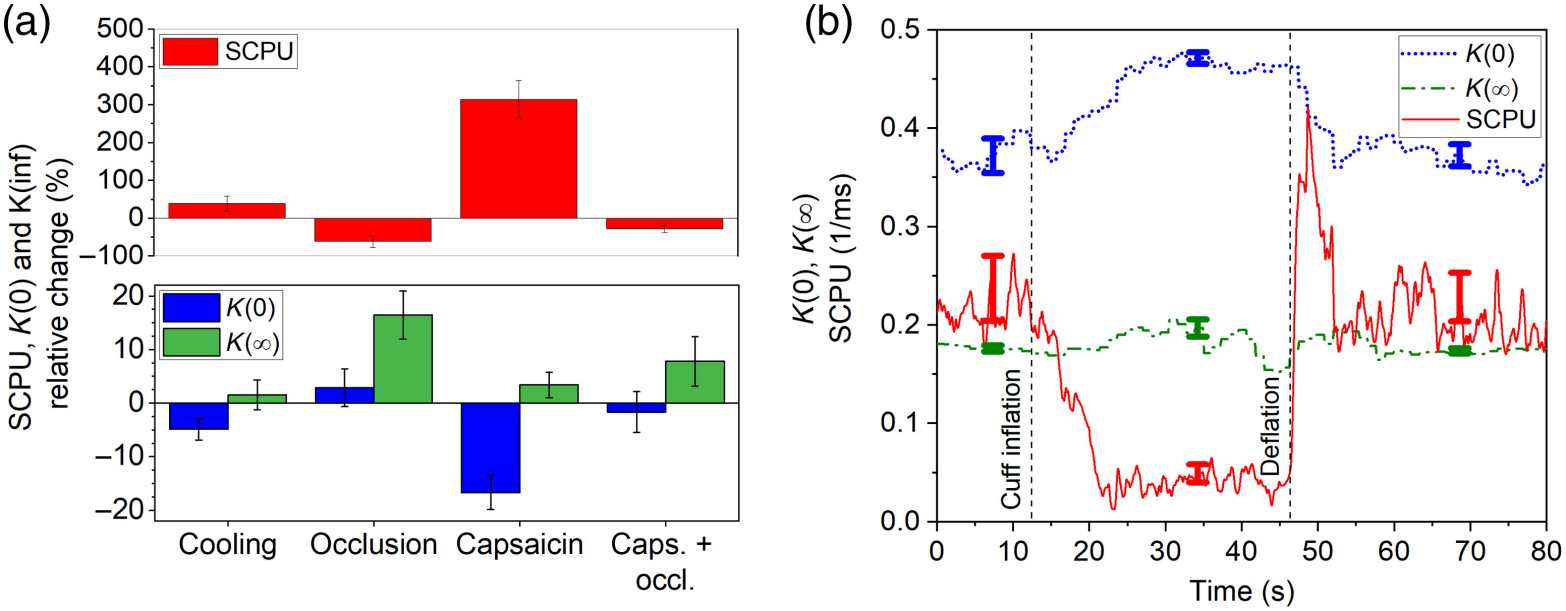
Variation of perfusion (SCPU) and the K(0), K(∞) asymptotic values of the contrast curve (a) for different stimuli and (b) time for occlusion applied to capsaicin treated skin.

In Fig. 3(a), the K(0), K(∞), and SCPU values are plotted for all applied stimuli in relation to their reference values obtained on the same area. For each measurement, the parameters are time-averaged for a time period of ∼15 to 20 s in case of occlusion, and 30 s for all other recordings, the error bars correspond to the standard deviation of averages for all the measurements. The data show that all these parameters can significantly change depending on the stimulus. The two extremities were the case of capsaicin and the occlusion. When applying capsaicin, the static contribution only slightly changed with significant decrease of the dynamic component and that of K(0). The occlusion resulted in a significant increase of the static component and a moderate decrease of the dynamic part, whereas the K(0) remained practically unchanged. During the reperfusion phase after cuff release, changes in the perfusion were so quick that our system could not accurately follow the changes in the asymptotes of the contrast curve. When occlusion was applied to the capsaicin treated arm, as a result of their combined effect, the fitting parameters approached the reference values. Figure 3(b) shows an example on time dependence of the parameters K(0), K(∞), and the perfusion when occluding the capsaicin treated arm, error bars indicate the standard deviation of the fluctuation for the corresponding evaluation period. The oscillations in the K(∞) value of the occluded arm could be caused by the involuntary twitches of the arm, which decrease the contrast at long-exposure time, whereas the short-exposure time images are less affected. Since a five points rolling average was used for the contrast values at a given exposure times, the overshoot at the beginning of the reperfusion is damped in the data curve. The absorption coefficients of hemoglobin and oxy-hemoglobin are the same at the 785 nm wavelength used in our system, the scattering properties are not expected to be influenced by the oxygenation of the blood. The intensity of the backscattered depolarized light reaching the camera was measured for the different stimuli; however, its variation was within a few percent ranges and did not show any tendentious correlation with the dynamic and static components of the contrast curve, nor with the measured perfusion.

**Fig. 4:**
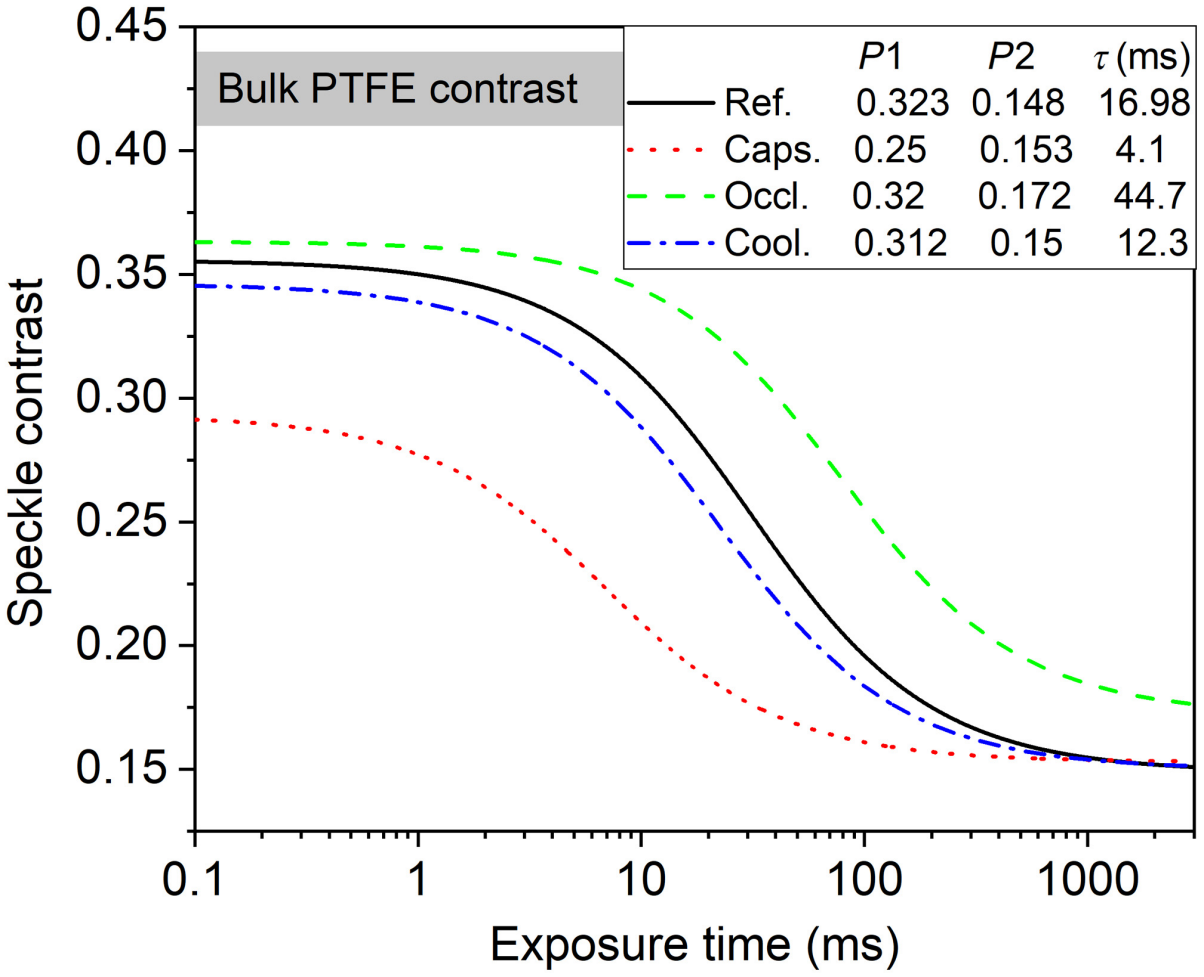
K(T) curves for different stimuli compared to the reference measured on the same skin area. Legend indicates the corresponding fitting parameters.

Figure 4 shows examples of the behavior of the K(T) curves of the monitored tissue before (marked as reference) and after the application of the stimuli on the same area. The curves were simulated using the fitting parameters obtained by continuous MESI and averaged for the corresponding stimuli. The contrast measured on a Teflon bulk also differs from the K(0) contrasts measured on the skin, indicating different scattering properties of the living tissue and the Teflon phantom often used for system calibration.

In earlier measurements on tissue phantoms, an increase of K(∞) and a decrease of K(0) were found when the scattering coefficient of the covering layer was significantly smaller than that of the emulsions (i.e., the average scattering coefficient decreased).^14^ Contrary to that, when the emulsion or skin surface was covered with PTFE layer having higher scattering coefficient (i.e., the average scattering coefficient increased),^20^ the K(0) has also increased. The latter is similar to the *in vivo* case, where the scattering coefficient of the skin is higher than that of blood.^31^ Changes in the average scattering and absorption coefficient of the skin with changes in perfusion have been shown in various studies.^32^^,^^33^

The shift of K(∞) observed during the measurements could simply be explained by the change in the fractional contribution of dynamically scattered light to the formation of the speckle images. The slight increase seen in the case of capsaicin was probably caused by the application of the cream on the skin surface since this change immediately observed, long before the capsaicin started to exert its effect on the perfusion. We suppose that the small increase of K(∞) observed in case of the cooling was also caused by the presence of moisture precipitated on the cold copper. In the case of arterial occlusion of the arm, the decrease in the perfusion is accompanied by the decrease of RBC concentration in the skin too.^34^^,^^35^ The decrease in RBC concentration results in a higher probability of the scattering on the non-moving components of the skin, as indicated by the higher K(∞) value.

For extremely low-exposure time, the differentiation between static and moving scattering center becomes meaningless; therefore, the altering K(0) indicates that the strongly different perfusion states are accompanied by different overall scattering properties of the monitored tissue. Decrease of K(0) was observed with cooling and capsaicin. While cooling usually leads to vasoconstriction, local cooling of the skin can result is vasodilation and increased perfusion,^36^ which is in agreement with our observations. Capsaicin also causes local vasodilation.^37^ The RBC enrichment in the subcutaneous region seems to strongly influence the scattering properties of the tissue, resulting in a decrease of the overall decorrelation factor.

**Fig. 5:**
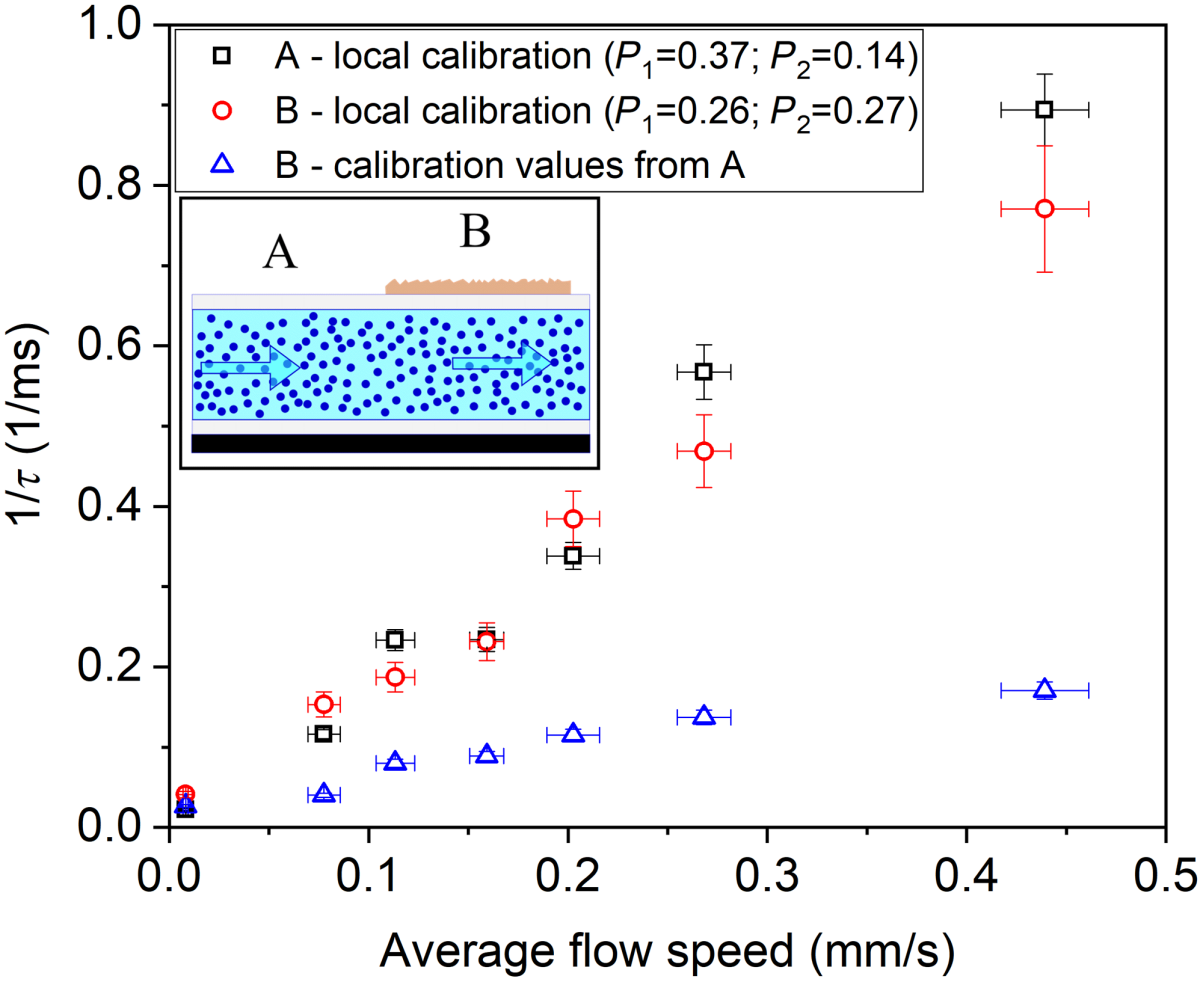
Flow speed dependence of reciprocal correlation time measured on microsphere emulsion for different scattering conditions (marked with A and B on the inset). Solid symbols correspond to the cases of proper local precalibration, open symbol data are obtained when calibration data of site A are applied on site B.

Since significant change in perfusion alters the dynamic range of the contrast curve, the monitoring of variations in the fitting/calibration parameters can increase the accuracy of the measurement of the correlation time τ. As a simple demonstration of the effect of changed scattering properties on the measurements, correlation times for the flow of microsphere emulsions were obtained for two different static scattering contributions. The application of PTFE above to opaque pipe in increased the K(∞) (from 0.14 to 0.27) and decreased the K(0) (from 0.40 to 0.38), which is to different degrees but a similar trend to the changes observed for some of the stimuli applied to the skin. Initially, $P_{1}$ and $P_{2}$ fitting parameters were locally determined by MESI without flow. Then the actual correlation times were calculated on the basis of 9 ms exposure time images, and the error bars are derived from the standard deviation of their frame-to-frame contrast and the uncertainty of flow speed measurement. Figure 5 shows reciprocal correlation time obtained on the regions with the different scattering properties (marked as A and B on the inset) and nearly equal and linearly dependent on the average flow speed if proper local calibration was used. However, when fitting parameters obtained on an uncovered pipe were applied for the PTFE covered region, the 1/τ becomes considerably different. This clearly indicates that when perfusion change is accompanied with changes in scattering properties, the measured change in perfusion can be non-negligibly different from the real one if parameters of the fitted models are not adjusted. The shift of K(∞) of this model experiment is higher than those observed in case of the skin, the magnitude of the differences in the measured correlation time will actually depend on the monitored tissue and the measuring parameters.

**Fig. 6:**
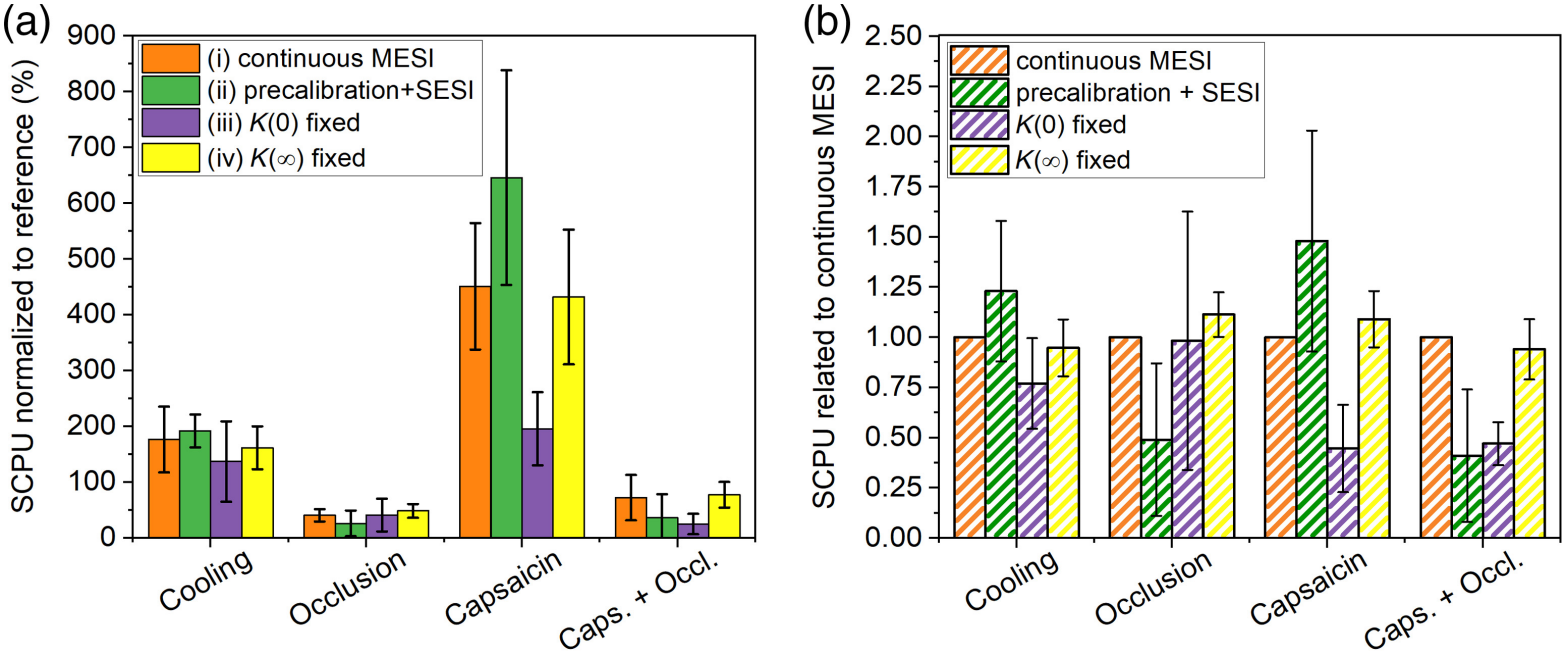
SCPU obtained in case of different stimuli with different evaluation methods normalized to reference measured on the same area before (a) the stimulus and (b) SCPU related to corresponding continuous MESI value.

As the temporal resolution of continuous MESI (which could adapt to different scattering properties) is relatively low as compared to SESI, different combined measurement approaches were used to speed up the measurements by enhancing the rate of the reciprocal correlation time (perfusion unit) calculations and the results were compared. The combined methods were based on a precalibration [obtaining the K(0) and K(∞) asymptotes] by means of MESI measurements on the normal skin and using some of those values as constants for the further measurements. The following evaluation methods were used to determine the correlation time in case of the different stimuli: (i) continuous free variable fitting on contrast values for all the exposure time values (continuous MESI); (ii) fixing the fitting parameters [i.e., both the K(0) and K(∞) asymptotes] to the reference values and calculating τ from the contrast value measured at 9 ms exposure, which corresponds to the case of precalibration followed by higher speed single-exposure monitoring; (iii) fixing of the K(0) asymptote and applying two long exposure times (81 and 162 ms) to obtain τ; and (iv) fixing only the static contribution (K(∞)) and obtaining τ by fitting to the contrast data obtained with the short-exposure times (1, 3, and 9 ms). The 1 and 3 ms exposure times are close to the asymptotic range of the K(T) curve and help the monitoring of K(0), whereas the 9 ms falls in the steeper range and it contributes to the determination of τ. Figure 6(a) shows the SCPU values obtained by different evaluation methods normalized to the corresponding reference and averaged for all the measurements. Figure 6(b) shows the SCPU values related to the continuous MESI (considered as unit) for each evaluated recording.

It is clearly shown that the data obtained by method (ii), which corresponds to the case of multiexposure precalibration followed by a higher speed single-exposure monitoring, differs strongly as compared to the data obtained by method (i) continuous MESI: the perfusion is overestimated in case of cooling and capsaicin [when K(0) decreases due to the stimulus] and strongly underestimated in case of occlusion [when K(∞) increases]. Application of method (iii) resulted in discrepancy as compared to the continuous MESI especially in case of capsaicin, indicating that the results are strongly influenced by the shift of K(0) caused by the RBC enrichment of the tissue. Overall, the smallest standard deviation of the data (indicated by the error bars) and the best correspondence between the continuous MESI and the temporal resolution enhancing methods was obtained for method (iv), which can be attributed to the followings: $P_{1}$ is about twice as high as $P_{2}$ (ranges between 0.25 and 0.323 versus 0.148 and 0.172), while these contribute quadratically to the measured contrast [see Eq. (2)]. The contrast for the short-exposure times used for fittings (1, 3, and 9 ms) is less influenced by the variation of static scattering and allows the continuous monitoring of the strongly varying K(0). The number of selected exposure times could be further reduced using a very short-exposure time. For example, using an exposure time of 0.1 ms or less, where the K(T) curves are saturated (see Fig. 4) the image contrast would directly correspond to K(0) (taking into account the statistical fluctuation of the speckle).

## 4 Conclusions

By creating a wide range of perfusion states of the skin, we studied systematically the behavior of contrast-exposure time dependence, which is of a key importance to determine the blood perfusion. The results showed that arterial occlusion caused an increase in the fractional contribution of static scattering to the speckle formation, and the increase of RBC content in the tissue could significantly decrease the short-exposure time asymptote K(0) as observed when applying capsaicin. These indicate that strongly different perfusion states are accompanied by different overall scattering properties of the monitored tissue, and therefore, a LASCA system precalibrated based on initial K(0) determination (e.g., β calibration) can lose its validity due to changes in perfusion. Depending on the actual setup and the tissue being monitored, the induced errors may be negligible (within the uncertainty of the measurements). However, if the dynamic range of the contrast curve is narrowed by static scattering (e.g., skin) and the overall scattering characteristics strongly during the measurements, it is worth considering adjusting the calibration to increase the measurement accuracy.

Considering that MESI may offer higher accuracy than SESI in terms of the observed variation of scattering properties at a much lower temporal resolution, we tested different evaluation methods that could combine the advantages of the two methods with some compromises. The results showed that an initial full exposure time range MESI sequence used as a precalibration, followed by the application of only 2 to 3 short-exposure time values in the saturating and steeper range of the K(T) curve, could be a promising method. This allows the continuous monitoring of the strongly varying K(0) while supposing a permanent K(∞) value since the correlation time calculation is less influenced by this later parameter.

## Code, Data and Materials Availability

Data underlying the results presented in this paper may be obtained from the authors upon reasonable request and through a collaboration agreement.
